# Relationships between transmission of malaria in Africa and climate factors

**DOI:** 10.1038/s41598-022-18782-9

**Published:** 2022-08-23

**Authors:** Biseko Juma Mafwele, Jae Woo Lee

**Affiliations:** 1 Department of Physics, Inha University, Incheon, Republic of Korea; 2 Department of Mathematics, Physics, and Informatics, Mkwawa University College of Education (MUCE), Iringa, Tanzania

**Keywords:** Ecology, Diseases, Physics, Statistical physics, thermodynamics and nonlinear dynamics

## Abstract

The spread of malaria is related to climate change because temperature and rainfall are key parameters of climate change. Fluctuations in temperature affect the spread of malaria by lowering or speeding up its rate of transmission. The amount of rainfall also affects the transmission of malaria by offering a lot of sites suitable for mosquitoes to breed in. However, a high amount of rainfall does not have a great effect. Because of the high malaria incidence and the death rates in African regions, by using malaria incidence data, temperature data and rainfall data collected in 1901–2015, we construct and analyze climate networks to show how climate relates to the transmission of malaria in African countries. Malaria networks show a positive correlation with temperature and rainfall networks, except for the 1981–2015 period, in which the malaria network shows a negative correlation with rainfall.

## Introduction

Malaria is a disease that continuously claims human lives in Africa and the rest of the world, including India, Brazil, and some Asian countries^1^. Malaria is an endemic disease in some countries and is mainly transmitted from infected mosquito bites^2–5^. It is known that malaria prevails in most tropical countries, and that is why most African countries suffer the consequences. Weather and climate are the major factors that drive increases in malaria in different areas. The elements of climate, especially temperature and rainfall, are the major drivers of malaria transmission^6–8^. The influence of climate on the transmission of malaria has been noted in many studies, including that a rise in malaria infections is related to both moderate temperature^6,9–12^, and rainfall^13,14^.

Several studies show there is a direct relation between malaria transmission and climate change^15–18^. The spread of malaria depends on the amount of rainfall, since rainfall creates a lot of sites suitable for mosquitoes to breed in^19–21^. Therefore, the incidence of malaria increases as the amount of rainfall increases in each area. However, a high amount of rainfall does not have a great effect^13^. The spread of malaria depends on the amount of temperature change or variation^22–24^. The fluctuation of temperature below the average temperature acts to speed up the rate of parasite development, while the fluctuation of temperature below the average temperature acts to slow down the rate of parasite development^11,12^. It is reported that the temperature ranges from 20 to 30 °C are optimal temperature to favor development of malaria parasite^24^. Moreover, optimal temperatures and maximum temperatures are the levels that favor development of malaria parasites^25^. The climatic variables temperature and rainfall are expected to have the non-linearities relationship with malaria Incidences. Therefore, climatic variables are expected to have a positive correlation with the incidence of malaria. The relationship between spreading of malaria and climate change is analyzed by using the malaria incidences and change of the temperature and rainfall data over the years. The incidence of malaria in one country can relate to the incidence in nearby countries. We refer to such connections and relations as a network that shows interactions (connection) between (countries) nodes^25^. In this network nodes represent countries where the data was collected, and interactions or connections represent the link or edges. Two countries are connected if they share common features, or they certify certain condition.

A network is the simplest description of a set of interconnected entities, which we call nodes and their connections which we call links or edge^25^. In other words, network is the collection of points joined in pair by line where point and line are referred as node and link respectively. For the two nodes to be connected they must share common features or interest or satisfies certain conditions. The nodes which are connected to many neighbors this is called the node is densely connected while if the node is connected to fewer neighbors this is called sparsely connected. For undirected network, Degree is the total number of links connected to a node. Hub is the most highly connected node in the network or Hub is be simply defined as the node will high degree^25^.

Tsonis and Swanson reported on the complex networks of the surface temperature field for El Nino and La Nina years. They constructed networks based on the Pearson correlation coefficient and by assigning an ad hoc threshold^26^. Donges et al. proposed a method to construct climate networks from data generated by a dynamic spatial–temporal system. They calculated mutual information for the time series and generated a complex network assigning a threshold value^27^. Arizmendi and Barreiro studied the seasonality of atmospheric connectivity and inter-annual variations in the El Nino–Southern Oscillation. They set a threshold of 99 quantiles for the distribution of the cross-correlation coefficients in a surrogate time series. They observed strong variability in the connectivity of the climate networks in both tropical and extratropical regions^28^. Wang applied the complex network methodology to construct an ocean observation complex network based on continuous data from mesoscale eddies in the South China Sea^29^.

Malaria transmission depends on many factors, like climate, land use, economic growth, and deliberate interventions. Snow et al. reported on the prevalence of Plasmodium falciparum in sub-Saharan Africa from 1900 to 2015. They observed the transmission cycles of malaria and discussed the major interventions against this disease^30^. The impact of temperature on population of the mosquito vector was analyzed by the population dynamic model. The population of the mosquito vector are highly correlated to the temperature^31^. The global climate change and local land use influences on malaria risk by the relationship between temperature and malaria parasite development^14^. The compartmental model applied to the spreading of the malaria in South Africa including climatic factors such as rainfall and temperature^21^. The long-term decline of malaria prevalence reported by analyzing historical data of the malaria in sub-Sahara Africa^30^. The power-law distribution of connection degrees reported on regional weather system using the high-resolution satellite data. They observed the spatial configuration of significant synchronizations between extreme rainfall events^32^. The decline in malaria prevalence across Africa in the period from 1960 to 1984 are related to several factors such as health agendas, cheap efficacious drug, and drought period of the Sahel^33,34^.

We consider the changes in malaria spread over time in Africa by using complex network analysis. We generate multiplex networks from the time series for temperature, precipitation, and the incidence of malaria to show how transmission of malaria relate with climate factors especially temperature and rainfall.

## Malaria, temperature, and rainfall networks

**Figure 1:**
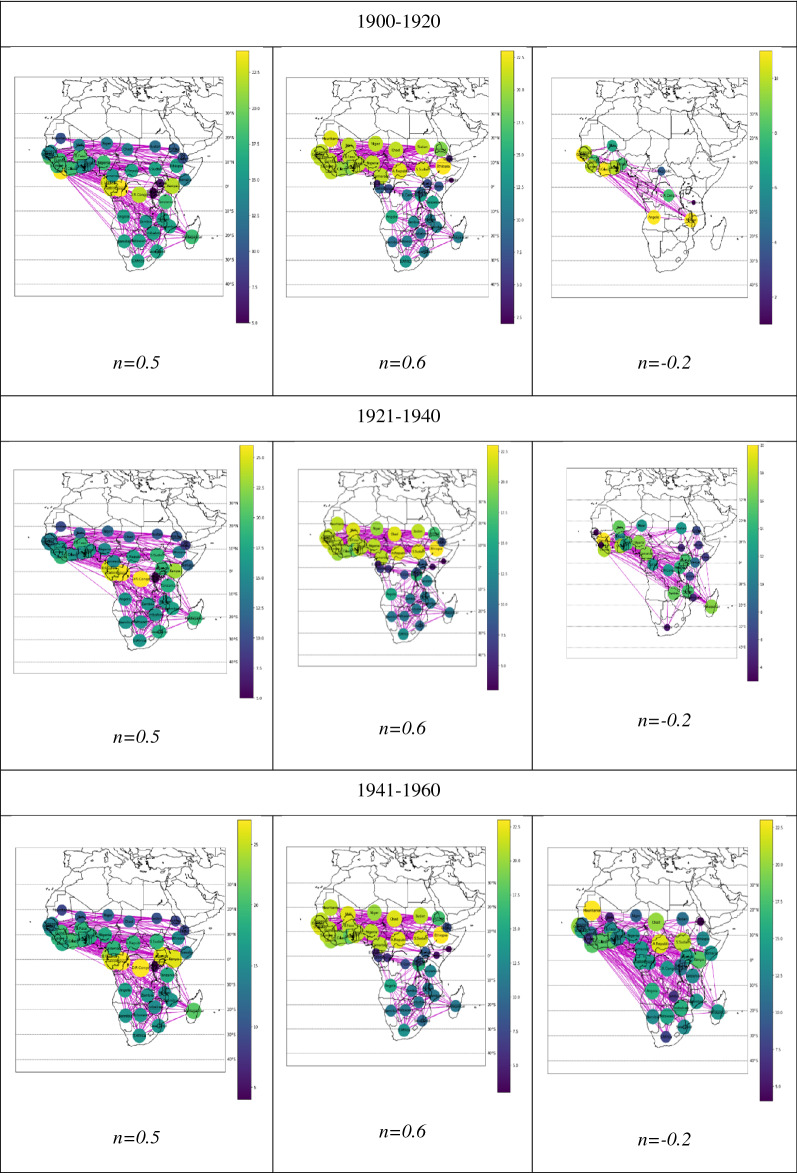

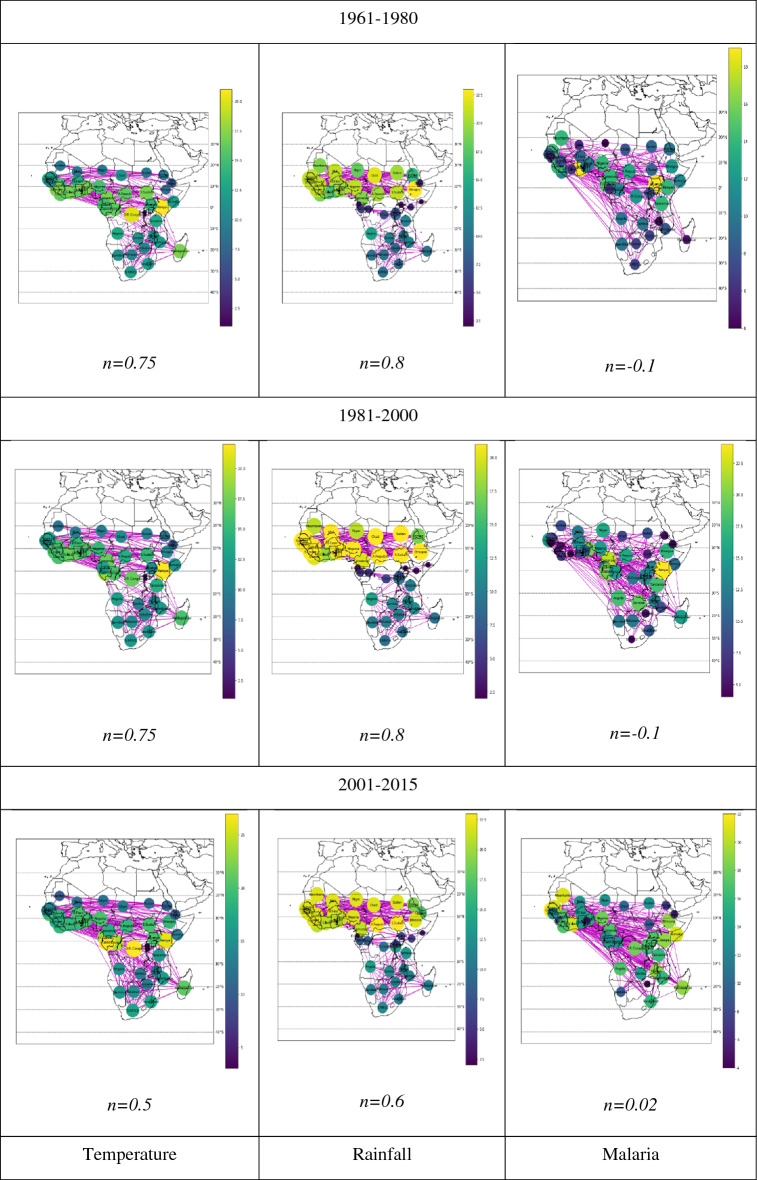
Networks connecting nodes with cross-correlation coefficients greater than the threshold value.

In Fig. 1, we present the networks generated from the cross-correlations among malaria, temperature, and precipitation time series. The color bar and color of the node indicate the degree of the node. In the rainfall networks for the northern parts of central, East, and West Africa, the nodes are highly connected to each other, except in Eritrea and Djibouti, which show different connectivity properties. In the southern part of Africa, the nodes are sparsely connected, and we observe similar connecting properties, except for the malaria networks from 1901 to 1920 and 1921 to 1940. In 1901–1920, the temperature network shows many hubs, except for Uganda, Rwanda, and Burundi, which are connected to fewer neighbor nodes. In this period, the malaria network has only 13 nodes, and contains a lot of isolated nodes along the equator.

In the network for 1941–1960, the nodes in southern parts of Africa in the temperature network and the malaria network are highly connected, whereas the nodes in the rainfall network are sparsely connected. The malaria network is densely connected in the northern part of Africa. The temperature network is highly connected between the equator and 10° N latitude, whereas above 10° N, those nodes are sparsely connected. In the malaria network, the nodes of some countries, such as South Sudan, Chad, the Central African Republic, and Mauritania, are densely connected to neighbor nodes. However, countries such as Eritrea, Mali, and Sierra Leone are sparsely connected, while the remaining nodes are highly connected to the neighbor nodes. Along the equator, the temperature network exhibits many hubs. The nodes in the temperature network are densely connected except for Rwanda and Burundi, which are sparsely connected to each other. In the rainfall network, the nodes along the equator are sparsely connected. But in the malaria network, the nodes along the equator are highly connected.

Consider the temperature network from 1981 to 2000. In the southern part of Africa and above 10° N, the nodes are sparsely connected. Between the equator and 10°N, nodes are densely connected to neighbor nodes. Along the equator, the network shows a decrease in the number of hubs, where in this period the temperature network has only one hub, namely Kenya. The nodes in the rainfall network from 1981 to 2000 are densely connected to neighbor nodes in the northern part of Africa but are sparsely connected to neighbor nodes in the southern part of Africa. Moreover, nodes along the equator are sparsely connected to neighbor nodes. The malaria network for the 1981–2000 period is sparsely connected to the neighbor nodes in West Africa, southern Africa, and a few central countries. Along the equator, the malaria network shows many hubs where Kenya is highly connected to neighbor nodes. The temperature network and malaria network in this period show the same properties along the equator.

The changes in the temperature network and rainfall network for each time interval are because the time series are correlated with climate change. The temperature network shows the presence of hub properties around the equator. In the southern part of Africa, nodes of the temperature network are highly connected between 0° and 10° N, and from 10 and 20° N. Moreover, the temperature network shows instability. In the rainfall network, nodes in the northern part of Africa are densely connected. In the southern part of Africa and along the equator, nodes are sparsely connected. Moreover, the rainfall network shows stability. In the malaria network, nodes in the southern part of Africa are sparsely connected; nodes in East Africa are highly connected, whereas, in West Africa, nodes are densely connected. Along the equator, the malaria network shows an increase in node connectivity; not only that, but the network also exhibits a hub property in the time intervals of 1961–1980 and 1981–2000. Moreover, the malaria network shows not only expansion but also stability. Although malaria is declining by various factors like health progress, cheap drugs, and education, we observe that the malaria time series is highly correlated to the time series of temperature and rainfall. We observe the correlation among them through three complex networks.

We obtained three threshold networks: temperature, precipitation, and incidence of malaria. The color bars indicate the magnitude of the degree in the network. The threshold parameter $n$ is given as $r_{ij} = \overline{r} + n\sigma$ where $r_{ij}$ is the cross-correlation coefficient between two nodes $i$ and *j,* $\overline{r}$ is an average, and ${\upsigma }$ is the standard deviation of the cross-correlation coefficients. We use Python 3.70, numpy for mathematical functions and random number generator, pandas for data analysis and manipulations, networkx for creation, manipulation, and studying the structure of the complex network, matplotlib for visualization and plotting graph and base map for map projection and visualization of geographic information.

## Network correlation

**Figure 2:**
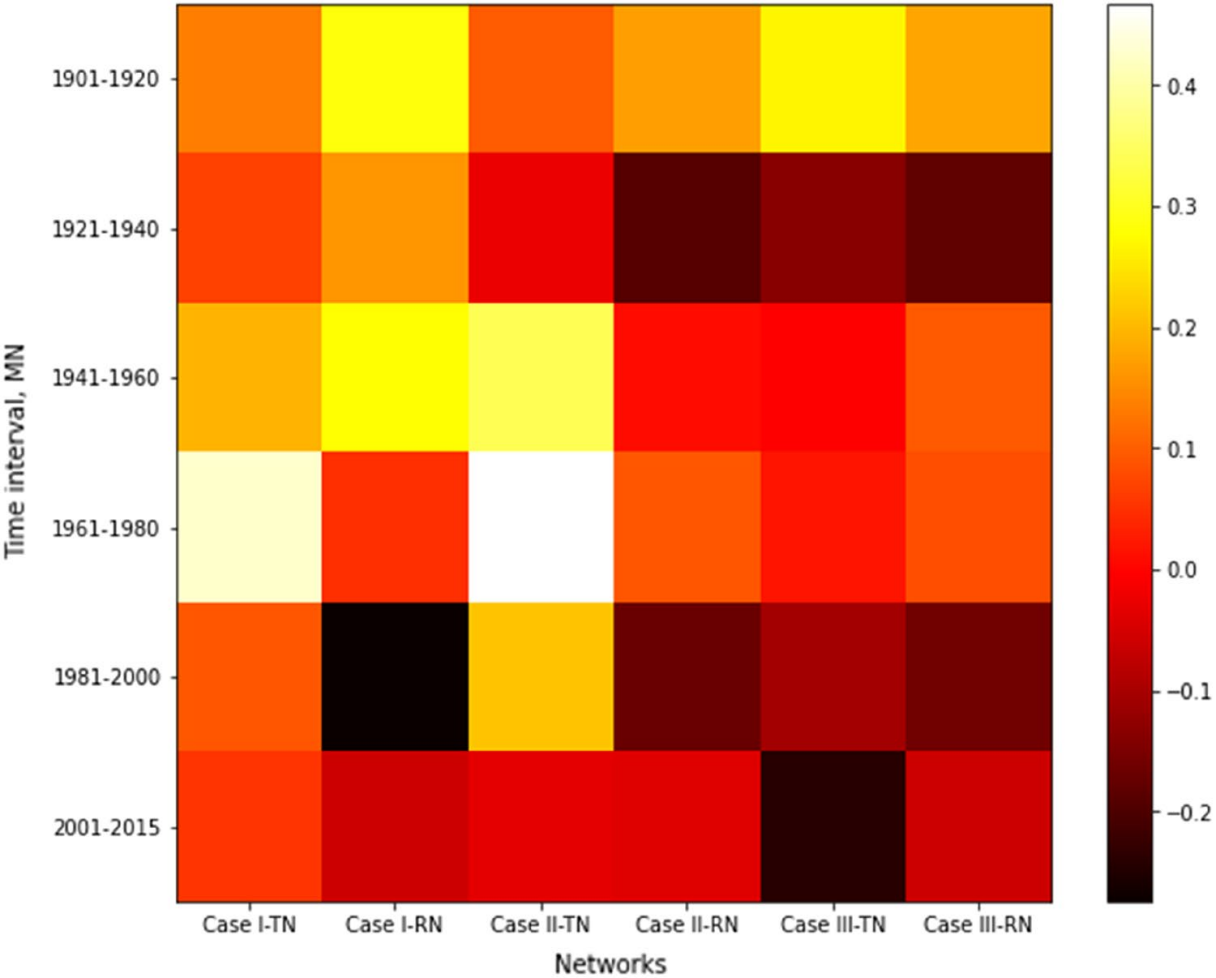
Degree correlations between three networks: temperature network (TN), rainfall network (RN), and incidence of malaria network (MN).

Consider the correlation between two networks. We calculate the degree of each node in the networks. Then, we estimate the normalized Pearson correlation coefficients based on the degrees between the two networks. If two nodes have high values, compared to the average value of the degree for each network, they are positively correlated with each other. However, if the degree of a node in a network is greater than the average degree, and the degree of the same node in other network is less than the average degree, they are negatively correlated. The quantitative relationship between the malaria networks, temperature networks, and rainfall networks is shown in Table S4. Figure 2 shows the risk map how the temperature network, the rainfall network, and the malaria network have related to each other. Case I in Fig. 2 describes the value of $n$ in the corresponding network where the values for $n$ are 0.5, 0.6, and − 0.2 for temperature, rainfall, and malaria networks, respectively. In the same fashion, Case II in Fig. 2 describes values of $n$ at 0.75, 0.8, and − 0.1 for temperature, rainfall, and malaria networks, respectively. In the same manner, Case III in Fig. 2 describes values of $n$ at 1.0, 1.0, and 0.1 for temperature, rainfall, and malaria networks, respectively.

When threshold value $n$ is low, as in Case I, we observe that the network for the incidence of malaria shows a positive correlation with temperature and rainfall networks, except in the rainfall network after the year 1981. The temperature networks are negatively correlated to the rainfall network and positively correlated to the malaria networks regardless of the period. When we increase the threshold value $n$, the networks become much sparser. In Case III, malaria networks negatively correlate to the temperature network, except for the 1901–1920 and 1961–1980 time periods. The correlation between malaria and rainfall networks alternates between positive and negative. Temperature networks are positively correlated to rainfall networks. In the most recent period (2001–2015), the malaria network negatively correlates to both temperature and rainfall networks in Case II and Case III.

We observe that fluctuations in the temperature network influence change in the malaria network. On the other hand, change in the rainfall network shows a slight relationship to change in the malaria network. Furthermore, from the correlation coefficient values for the degree, we see that the malaria network relates to the temperature and rainfall networks. Therefore, according to the network results such as the relationship between node and edge, the correlation coefficient of the networks all show that malaria transmission depends on climatic elements such as temperature and rainfall. We need to further extend the analysis of the single layer to an analysis of multilayer networks in a future study.

## Methods and materials

### Data

We used temperature data, rainfall data, and data on the incidence of malaria collected from 1901 to 2015 for 43 African countries to construct networks to determine the relationships between transmission of malaria and climate change elements, especially temperature and rainfall. Data resolution is given by the latitude and longitude of the capital city for every country in Africa. Temperature and rainfall data are provided in terms of monthly averages in the country wise. The nodes in the network represent the country, and the edges in the network represent the relationship between countries. We collected malaria data from Harvard Dataverse^35^ and the world malaria report from the WHO^31^. Data for temperature and rainfall were obtained from the Climate Change Knowledge Portal of the World Bank Group^36^.

### Network generation and analysis

The networks were constructed by using the threshold method where the network depends on the mean, standard deviation, and the real number ($n$) used to control the features of the network. Therefore, data for temperature, rainfall, and the incidence of malaria were divided into six groups mostly comprising ranges of 20 years (1900–1920, 1921–1940, 1941–1960, 1961–1980, 1981–2000) as well as the period from 2001 to 2015. The missing data in Malaria incidence data are filled by the average amount of malaria incidence collected per year.

In Table S1, a malaria report from the World Health Organization shows that the rate of death is directly proportional to the incidence of malaria^35^. The death toll in Africa from malaria is about 98% of world deaths from malaria. Such deaths in African regions decrease thanks to efforts the WHO, governments, and the private sector have been conducting to prevent them. Weather and climate are among the factors that drive increases in malaria infections in different areas.

We consider networks based on the threshold method (see the “Methods and Materials” section below). First, we fill the missing malaria incidence data, and we calculate normalized Pearson correlation coefficients of three-time series between two countries. Then, we obtain a correlation matrix for the countries. We estimate the average value of the correlation coefficients from the time intervals 1901–1920, 1921–1940, 1941–1960, 1961–1980, 1981–2000, and 2001–2015 for three time series: temperature, rainfall, and incidence of malaria. We summarize the averages and standard deviations of the correlation coefficients, as shown in Table S2. The mean values from the correlation in temperature are high, compared to those for rainfall and the incidence of malaria. The standard deviations in temperature and rainfall are large, but the standard deviation for the incidence of malaria is small.

We chose an ad hoc threshold value of the correlation coefficients to generate sparse networks. The characteristic values for $n$ of the threshold are given in Table S3. We consider three types of thresholds in order to observe changes in the networks according to the threshold.

Let us define the normalized variance of each time series. We considered time series $T_{i} \left( t \right)$, $M_{i} \left( t \right)$, and $R_{i} \left( t \right)$ in country $i$ for temperature, the incidence of malaria, and rainfall, respectively. We define normalized variance as

1 $$r_{ij} = \frac{{x_{i} \left( t \right)x_{j} \left( t \right) - x_{i} \left( t \right)x_{j} \left( t \right)}}{{\sigma_{i} \sigma_{j} }}$$

where $x_{i} \left( t \right)$ = $T_{i} \left( t \right)$, $M_{i} \left( t \right)$, $R_{i} \left( t \right)$. We obtained a Pearson correlation matrix for each time series as follows:

2 $$R_{S} = \left[ {\begin{array}{*{20}c} {r_{11} } & \cdots & {r_{1N} } \\ \vdots & {r_{ij} } & \vdots \\ {r_{N1} } & \cdots & {r_{NN} } \\ \end{array} } \right]$$

where $$S = T, M, R$$.

We calculated the average value, $\overline{r }$, and the standard deviation, $\sigma$, for the correlation coefficients of the matrix. We applied the threshold method to generate a sparse network from the correlation matrix. Two countries are connected in the correlation network if and only if the value of the correlation coefficient is greater than, or equal to, the threshold value:

3 $$r_{{ij}} = \left\{ {\begin{array}{*{20}c} 1 & {{\text{if}}\;r_{{ij}} \ge \bar{r}{\text{ + n}}\sigma } \\ 0 & {{\text{otherwise}}} \\ \end{array} } \right.$$

where $r_{ij}$ is the correlation coefficient between two countries, and $n$ is an element of real numbers ($n \in {\mathbb{R}}$). The value of $n$ determines whether the network is sparsely or densely connected.

We use Python programming language, packages, numpy for mathematical functions and random number generator, pandas for data analysis and manipulations, networkx for creation, manipulation, and studying the structure of the complex network, matplotlib for visualization and plotting graph and base map for map projection and visualization of geographic information.

## Supplementary Information

Supplementary Information.

## Data Availability

Raw data were obtained from publicly available sources like Harvard Dataverse and the WHO’s world malaria report. We collected malaria data from Harvard Dataverse^35^ and the world malaria report from the WHO^31^. Data for temperature and rainfall were obtained from the Climate Change Knowledge Portal of the World Bank Group^36^.
