# Supplementary material for: Relationships between transmission of malaria in Africa and climate factors
Source: Sci Rep. 2022 Aug 23;12:14392. doi: 10.1038/s41598-022-18782-9 (PMC9399114; doi:10.1038/s41598-022-18782-9)
Supplement: Supplementary file 1 — Supplementary Information. [file 41598_2022_18782_MOESM1_ESM.docx]

Supplementary Information

1. Malaria report
2. Mean and standard deviation of the correlation
3. Threshold parameters
4. Degree correlations between three networks
5. Malaria report

We summarize the malaria report published by WHO (World Health Organization) in Table S1. The death tolls by malaria are creasing over the time.

Table S1 Malaria report summary

| Year | Global cases  (million) | Global deaths  (thousand) | Africa cases  (million) | Africa Deaths  (thousand) |
| --- | --- | --- | --- | --- |
| 2000 | 238 | 697 | 204 | 680 |
| 2001 | 244 | 700 | 210 | 685 |
| 2002 | 239 | 698 | 207 | 685 |
| 2003 | 244 | 681 | 211 | 672 |
| 2004 | 248 | 708 | 214 | 706 |
| 2005 | 247 | 662 | 211 | 653 |
| 2006 | 242 | 675 | 211 | 667 |
| 2007 | 241 | 644 | 211 | 637 |
| 2008 | 240 | 599 | 211 | 590 |
| 2009 | 246 | 572 | 215 | 569 |
| 2010 | 247 | 546 | 215 | 542 |
| 2011 | 239 | 505 | 211 | 501 |
| 2012 | 234 | 481 | 209 | 477 |
| 2013 | 225 | 451 | 205 | 454 |
| 2014 | 217 | 440 | 197 | 435 |
| 2015 | 218 | 422 | 199 | 418 |
| 2016 | 226 | 403 | 205 | 395 |
| 2017 | 231 | 396 | 212 | 388 |
| 2018 | 228 | 389 | 212 | 385 |
| 2019 | 229 | 387 | 215 | 384 |

## Mean and standard deviation of correlation

Table S2 shows the values of mean and standard deviation of correlation coefficient in temperature, rainfall, and malaria in each time interval.

Table S2 Mean and standard deviation values of correlation coefficient in temperature, rainfall, and malaria in each time interval.

| Time interval | Temperature | | Rainfall | | Malaria | |
| --- | --- | --- | --- | --- | --- | --- |
|  | Mean | STD | Mean | STD | Mean | STD |
| 1901-1920 | 0.2214 | 0.5161 | 0.0413 | 0.6021 | -0.0028 | 0.0430 |
| 1921-1940 | 0.2458 | 0.5187 | 0.0436 | 0.6231 | 0.0092 | 0.0901 |
| 1941-1960 | 0.2847 | 0.5400 | 0.0476 | 0.6219 | 0.0113 | 0.0971 |
| 1961-1980 | 0.2924 | 0.5342 | 0.0481 | 0.6252 | -0.0001 | 0.0670 |
| 1981-2000 | 0.2825 | 0.5325 | 0.0571 | 0.6279 | 0.0031 | 0.0652 |
| 2001-2015 | 0.2743 | 0.5242 | 0.0503 | 0.6213 | 0.0103 | 0.0871 |

## Threshold parameters

In this study the values of $n$ are not uniform to all networks since networks do not show the same properties at identical values of threshold. For example, temperature, rainfall, and malaria network become sparse and denser at different values of $n$. Therefore, the values of $n$ depend on the kind of the network. Table S3 shows various values of $n$ for temperature, rainfall, and malaria network.

Table S3 Values of $n$ for temperature, rainfall, and malaria network.

| Type | Type I | Type II | Type III |
| --- | --- | --- | --- |
| Temperature network | 0.5 | 0.75 | 1.0 |
| Rainfall network | 0.6 | 0.8 | 1.0 |
| Malaria network | -0.2 | -0.1 | 0.1 |

1. Degree correlations between three networks

Table S4 shows how the temperature network, the rainfall network, and the malaria network have related to each other. Case I in Table S4 describes the value of $n$ in the corresponding network where the values for $n$ are 0.5, 0.6, and -0.2 for temperature, rainfall, and malaria networks, respectively. In the same fashion, Case II in Table 1 describes values of $n$ at 0.75, 0.8, and -0.1 for temperature, rainfall, and malaria networks, respectively. In the same manner, Case III in Table 1 describes values of $n$ at 1.0, 1.0, and 0.1 for temperature, rainfall, and malaria networks, respectively.

Table S4 Degree correlations between three networks: temperature, rainfall, and incidence of malaria.

| Time interval | Network | Case I | | Case II | | Case III | |
| --- | --- | --- | --- | --- | --- | --- | --- |
|  |  | TN | RN | TN | RN | TN | RN |
| 1901-1920 | MN | 0.136 | 0.285 | 0.098 | 0.172 | 0.268 | 0.180 |
| 1921-1940 | MN | 0.069 | 0.162 | -0.023 | -0.190 | -0.134 | -0.178 |
| 1941-1960 | MN | 0.197 | 0.277 | 0.341 | 0.010 | -0.004 | 0.095 |
| 1961-1980 | MN | 0.429 | 0.049 | 0.467 | 0.091 | 0.021 | 0.083 |
| 1981-2000 | MN | 0.091 | -0.274 | 0.215 | -0.169 | -0.105 | -0.161 |
| 2001-2015 | MN | 0.054 | -0.057 | -0.032 | -0.037 | -0.24 | -0.057 |
